# Factors associated with suicidal ideation in healthcare personnel: a systematic review

**DOI:** 10.3389/fpsyg.2025.1717231

**Published:** 2025-12-29

**Authors:** Carlos Fernández-Peinado García, María Cantero-García, Daniel Dorta-Afonso, María Rueda-Extremera

**Affiliations:** 1 Facultad de Psicología y Ciencias de la Salud Madrid, Universidad a distancia de Madrid (UDIMA), Madrid, Spain; 2 Dpto. de Economía y Dirección de Empresas, Universidad Las Palmas de Gran Canaria (ULPGC), Las Palmas de Gran Canaria, Spain

**Keywords:** suicide, suicidal ideation, healthcare professionals, risk factors, protective factors, resilience

## Abstract

**Aim:**

This paper investigates suicidal ideation among healthcare professionals, a growing concern that affects their mental well-being and the quality of healthcare delivery. The study aims to identify key risk factors, such as work-related stress, exposure to death, and lack of institutional support, that contribute to suicidal ideation in this population. It also explores protective factors, including resilience, social support, and institutional resources, that may mitigate these risks.

**Method:**

A systematic review was conducted on studies published between 2020 and 2024. The literature search spanned databases such as PubMed, Scopus, Web of Science, PsycINFO, Dialnet, and Scielo. The review followed the PRISMA guidelines to ensure thoroughness and transparency in study selection. To assess the quality of the included studies, standardized tools like the Newcastle–Ottawa Scale were applied.

**Results:**

The review identified that the COVID-19 pandemic has intensified factors leading to suicidal ideation among healthcare professionals, with a notable increase in prevalence during this period. Identified risk factors included high levels of occupational stress, frequent exposure to death and suffering, and insufficient institutional support. Conversely, protective factors like resilience, social support, and access to institutional resources were found to reduce susceptibility to suicidal ideation.

**Conclusion:**

The findings highlight an urgent need for comprehensive prevention strategies and support programs targeting healthcare personnel. Recommendations for interventions span individual, organizational, and public policy levels. Enhancing resilience and providing institutional support could be crucial steps in reducing the incidence of suicidal ideation in this vulnerable group, ultimately improving both their mental health and the quality of healthcare services.

## Highlights

- Critical focus: Investigates the rising prevalence of suicidal ideation among healthcare professionals, emphasizing the impact on mental well-being and healthcare quality.
- Risk factors identified: Examines key risk factors, including work-related stress, exposure to death and suffering, and insufficient institutional support, that contribute to suicidal ideation.
- Protective factors: Explores protective elements such as resilience, social support, and access to institutional resources that can mitigate the risk of suicidal thoughts.
- Systematic review methodology: Utilizes a rigorous systematic review approach, analyzing studies published between 2020 and 2024, following PRISMA guidelines for transparency and thoroughness.
- Impact of COVID-19: Highlights how the COVID-19 pandemic has intensified risk factors for suicidal ideation among healthcare workers, with a notable increase in prevalence during this period.
- Urgent recommendations: Calls for comprehensive prevention strategies and support programs at individual, organizational, and policy levels to address mental health challenges in healthcare settings.
- Policy implications: Suggests that enhancing resilience and providing institutional support are crucial steps in reducing suicidal ideation, ultimately improving both healthcare personnel’s mental health and the quality of care they deliver.

## Introduction

Suicidal behavior is a complex and multifaceted phenomenon that includes actions ranging from ideation to completed suicide. Recent studies have shown that the prevalence of suicidal ideation among healthcare professionals is significantly higher than in the general population, highlighting the urgent need to address this issue from a public health perspective (Cano-Langreo et al., 2014; Olfson et al., 2023). The high prevalence of suicidal ideation among healthcare professionals has serious repercussions for their lives and well-being, as well as for the quality of the healthcare system (Olfson et al., 2023). The health crisis caused by the COVID-19 pandemic has exacerbated stress and burnout levels in this group (Linzer et al., 2023).

However, suicidal ideation does not manifest uniformly across all healthcare professionals. Evidence indicates that physicians tend to exhibit higher rates of suicidal ideation compared to other health workers, often due to professional isolation, high responsibility, and stigma surrounding mental health help-seeking (Hawton et al., 2004). In contrast, nurses are more prone to suicidal ideation linked to emotional exhaustion, moral distress, and limited institutional support (Davidson et al., 2020). Medical residents and interns represent another highly vulnerable group, as long working hours, sleep deprivation, and intense performance pressure contribute to psychological distress (Shanafelt et al., 2022). Meanwhile, allied health professionals, such as technicians and therapists, may experience suicidal thoughts associated with job insecurity and lack of recognition. These distinctions emphasize that suicidal ideation varies depending on professional role, workplace conditions, and access to support resources, underscoring the need for targeted preventive strategies. Understanding these role-based differences is essential when analyzing the broader psychological and occupational risk factors contributing to suicidal ideation.

Among the factors that increase the risk of suicidal ideation in healthcare professionals are mental disorders, such as depression and anxiety, as well as traits like perfectionism and excessive self-criticism. These professionals face internal pressure to meet high standards, which, combined with constant work-related stress, deteriorates their mental well-being. However, there are protective factors that can mitigate this risk. Resilience, understood as the ability to adapt positively to adversity, is crucial in protecting against suicidal thoughts. García-Iglesias et al. (2022) indicate that healthcare workers with high levels of resilience are less likely to experience suicidal ideation, even in stressful contexts.

From a practical standpoint, understanding the causes and risk factors associated with suicidal ideation in healthcare professionals can help design specific interventions. Measures such as promoting resilience and implementing psychological support programs have proven effective (McCann et al., 2013). Additionally, therapies such as interpersonal suicide therapy (Joiner, 2005) have proven effective in reducing suicidal ideation, emphasizing the importance of personal relationships and the individual’s sense of belonging. Social interactions not only influence the development of suicidal thoughts but can also lead to their realization through feelings of isolation and the self-perception of being a burden to others.

### Mental health conceptualization

According to the World Health Organization (WHO), this mental health encompasses emotional, psychological, and social aspects that enable individuals to develop their potential, manage daily stress, and make healthy decisions (WHO, 2014).

In the context of healthcare professionals, an optimal state of mental health is essential for them to perform their duties effectively and empathetically. When mental health is compromised, the risk of chronic stress, burnout, and other mental disorders increases, affecting the overall well-being of staff and their ability to balance their professional and personal lives (Shanafelt and Noseworthy, 2017).

#### Suicidal ideation and suicide

Suicidal ideation is a sign of severe psychological distress and a significant risk factor (Sobregrau et al., 2022). A suicide attempt is a clear indicator that the person needs immediate intervention and psychological support. Although these attempts do not always lead to death, they can cause significant physical and emotional harm (Perlis, 2023). Finally, completed suicide represents a tragedy both for the individual who carries it out and for their close ones, and it is the extreme outcome of an unresolved mental health crisis. In healthcare professionals, a completed suicide reflects the profound impact of adverse working conditions and the lack of adequate support (Awan et al., 2022).

Recognizing the manifestations and symptoms of suicidal ideation in healthcare professionals is necessary for early intervention (Padmanathan et al., 2023). Some of the most common symptoms were included in Table 1.

**Table 1:** Symptoms of suicidal ideation in healthcare professionals.

Symptom category	Description
Behavioral changes	Decreased work performance, frequent absenteeism, or lack of interest in activities previously enjoyed
Social isolation	Avoidance of interaction with colleagues, friends, and family, which may serve as a mechanism to hide distress or stem from feelings of being misunderstood
Hopelessness and helplessness	Unease about the future or belief that circumstances will never improve, often accompanied by the belief that suicide is the only solution to their situation
Expression of suicidal thoughts	Verbalization of suicidal thoughts, talk about death, or expression of a lack of meaning in life. May also manifest subtly through comments such as “wishing to rest forever” or “escaping everything”
Physical changes	Weight loss, insomnia or hypersomnia, and lack of self-care, reflecting a general deterioration in physical and emotional well-being

#### Risk factors

Healthcare professionals face multiple risk factors that affect their mental health and increase the likelihood of suicidal ideation. One of the most significant is occupational stress, which can arise from various sources, such as workload, constant exposure to death and suffering, and making critical decisions under pressure. The workload in the healthcare sector is often overwhelming, with long working hours, limited break time, and a high number of patients to attend to. This constant pressure can lead to physical and mental exhaustion, increasing the risk of chronic stress (Prasad et al., 2021). Additionally, professionals must maintain high levels of alertness and efficiency for extended periods, further deteriorating their well-being.

The constant confrontation with mortality and the helplessness in the face of insufficient resources or effective treatments can cause considerable psychological wear (Di Giuseppe et al., 2021). The difficulty in managing emotions related to these experiences can result in a continuous state of emotional tension. Moreover, the responsibility of making decisions that can determine a patient’s life or death, especially under uncertain conditions and time pressure, increases anxiety levels and fear of making mistakes (Butler et al., 2022).

Prolonged stress in the healthcare environment can lead to anxiety disorders, depression, and burnout, the latter characterized by emotional exhaustion, depersonalization, and a sense of reduced personal accomplishment (Padmanathan et al., 2023). Burnout is particularly prevalent among healthcare professionals and is considered a common precursor to suicidal ideation (Varela-Agra et al., 2025).

The work environment also influences the mental health of healthcare professionals. Adverse physical conditions, such as poorly ventilated workspaces, excessive noise, and inadequate lighting, can deteriorate workers’ well-being. Furthermore, a hostile social environment, with a lack of support among colleagues, workplace harassment, or ineffective management, generates high levels of stress and anxiety. The lack of open communication and social support contributes to isolation, increasing suicidal ideation (Davis et al., 2021).

The scarcity of resources and institutional support exacerbates these problems. In many healthcare settings, psychological support tools are limited or nonexistent; even when available, they are often insufficient or poorly integrated into the work routine (Härkänen et al., 2023). Finally, professionals face extremely high expectations, both self-imposed and imposed by society and institutions (Di Giuseppe et al., 2021). The pressure to maintain a high level of competence and provide quality care can be overwhelming, generating feelings of inadequacy or failure when outcomes do not meet expectations.

#### Protective factors

Healthcare professionals face challenges that can lead to suicidal ideation, but there are protective factors that can mitigate these risks and promote their mental well-being. Social and family support is fundamental; having a strong network of friends, family, and colleagues provides a sense of belonging and understanding, reducing social isolation and emotional fatigue (Danet, 2021; Acoba, 2024).

Moreover, resilience plays a crucial role in maintaining mental health. This characteristic allows individuals to adapt to adversity and recover from stressful experiences (García-Iglesias et al., 2022). Resilient healthcare professionals tend to use adaptive coping strategies and can maintain a positive outlook even in the face of challenges. Interventions aimed at strengthening resilience, such as mindfulness practices or cognitive-behavioral therapy, have demonstrated effectiveness in reducing symptoms of anxiety and depression among healthcare professionals (Ahmad et al., 2025) Providing healthcare professionals with adequate support systems, such as psychological counseling and peer support groups, fosters emotional well-being (Härkänen et al., 2023). Programs that promote mental health awareness and destigmatize seeking help encourage professionals to seek support when needed. The availability of mental health training and education on stress management and self-care strategies empowers professionals to take control of their mental well-being (Davis et al., 2021).

Organizational factors also play a significant role. A supportive work environment characterized by open communication, recognition, and constructive feedback positively influences mental health (Shanafelt and Noseworthy, 2017). Institutions that implement programs to promote work-life balance, stress management, and well-being can significantly reduce burnout and improve job satisfaction among healthcare professionals. Encouraging teamwork and camaraderie among colleagues fosters a sense of community that enhances mental well-being (Linzer et al., 2023).

Finally, engaging in self-care activities, such as physical exercise, hobbies, and relaxation techniques, is essential for maintaining mental health. Healthcare professionals should be encouraged to prioritize their own well-being and develop healthy coping strategies to deal with the demands of their work (Davis et al., 2021).

### Objectives

The primary purpose of this systematic review is to identify and examine the individual, workplace, and organizational factors associated with suicidal ideation among healthcare personnel. The specific objectives include:

- *Collecting and analyzing available evidence* regarding the prevalence of suicidal ideation among different groups of healthcare professionals, distinguishing between medical staff, nursing personnel, and other workers in the sector.
- *Determining the most relevant and predominant risk factors* contributing to suicidal ideation in this population, considering workplace aspects such as workload, long hours, institutional support, and chronic stress. Personal factors such as history of mental health issues, social support networks, and traumatic experiences will also be investigated, along with organizational factors including human resource policies and institutional culture that may impact employees’ mental health.
- *Evaluating the methodological quality of the included studies* to establish the robustness of the findings, utilizing standardized assessment tools and methods to identify biases, such as the PRISMA guidelines and the Newcastle-Ottawa tool. This analysis will highlight potential limitations and areas needing improvement in existing research.
- *Providing evidence-based recommendations* for the prevention and management of suicidal ideation among healthcare personnel. This will include suggestions for implementing wellness and psychological support programs, strategies to promote resilience, and the development of workplace policies prioritizing mental health and employee well-being.

### Research question

What personal, workplace, and organizational factors are linked to suicidal ideation in healthcare personnel?

## Method

To conduct this review comprehensively, taking into account the various elements that must be included, the PRISMA guidelines (Urrútia and Bonfill, 2010) were applied to ensure quality and transparency in the article selection process (Liberati et al., 2009). A systematic search was conducted to identify research on risk and protective factors associated with suicidal ideation in healthcare personnel between June and August 2024.

The selection parameters were based on the PICOS framework (Higgins and Green, 2011) and were established prior to the article selection (Table 2). Once the inclusion and exclusion criteria were drafted, a systematic search of the literature was conducted across databases such as Scopus, PubMed, Web of Science, PsycINFO, Dialnet, and Scielo, which are among the primary tools for locating scientific literature in health sciences. Table 3 presents a summary of the key criteria for the exclusion and inclusion of articles.

**Table 2:** PICOS system.

Parameters	Description
Participants (P)	Healthcare professionals (doctors, nurses, nursing assistants, and other healthcare workers)
Intervention (I)	The review will analyze risk and protective factors related to suicidal ideation and suicide, as well as various prevention and management strategies for suicidal ideation
Comparison (C)	Comparisons among different groups of healthcare professionals in relation to suicidal ideation Comparisons of intervention and prevention programs
Outcomes (O)	Incidence and prevalence of suicidal ideation Identification of risk and protective factors Effectiveness of interventions and support programs
Study Design (S)	Quantitative research articles

**Table 3:** Summary of inclusion and exclusion criteria.

Inclusion criteria	Exclusion criteria
Articles published in the last 5 years	Studies that do not include healthcare professionals
Articles written in English, Portuguese, or Spanish	Studies that focus on populations unrelated to the healthcare field
Articles with data on the variables: burnout, anxiety, stress, depression, resilience	Interventions that do not address suicidal ideation or the mental health of healthcare personnel
Quantitative studies	Studies without a comparison group or that do not include relevant comparisons for suicidal ideation among healthcare professionals
Studies with full access	Studies without full access or that are not written in English, Portuguese, or Spanish
Studies that include specific data on suicidal ideation among healthcare professionals	Research that is not psychological or addresses other issues is excluded

### Search

The search strategy was designed using a combination of Medical Subject Headings (MeSH) terms and free keywords, applying Boolean operators AND and OR to maximize the relevance and accuracy of the results. The search focused on identifying observational studies investigating suicidal ideation and associated risk factors among healthcare professionals. An example of the search strategy used in PubMed is as follows: (suicid* OR “suicidal ideation” OR “suicidal thoughts”) AND (“healthcare personnel” OR “health professionals” OR “health workers” OR medic* OR nurs* OR physician*) AND (“risk factors” OR “associated” OR “correlated” OR “predictive”).

#### Publication selection

**Figure 1:**
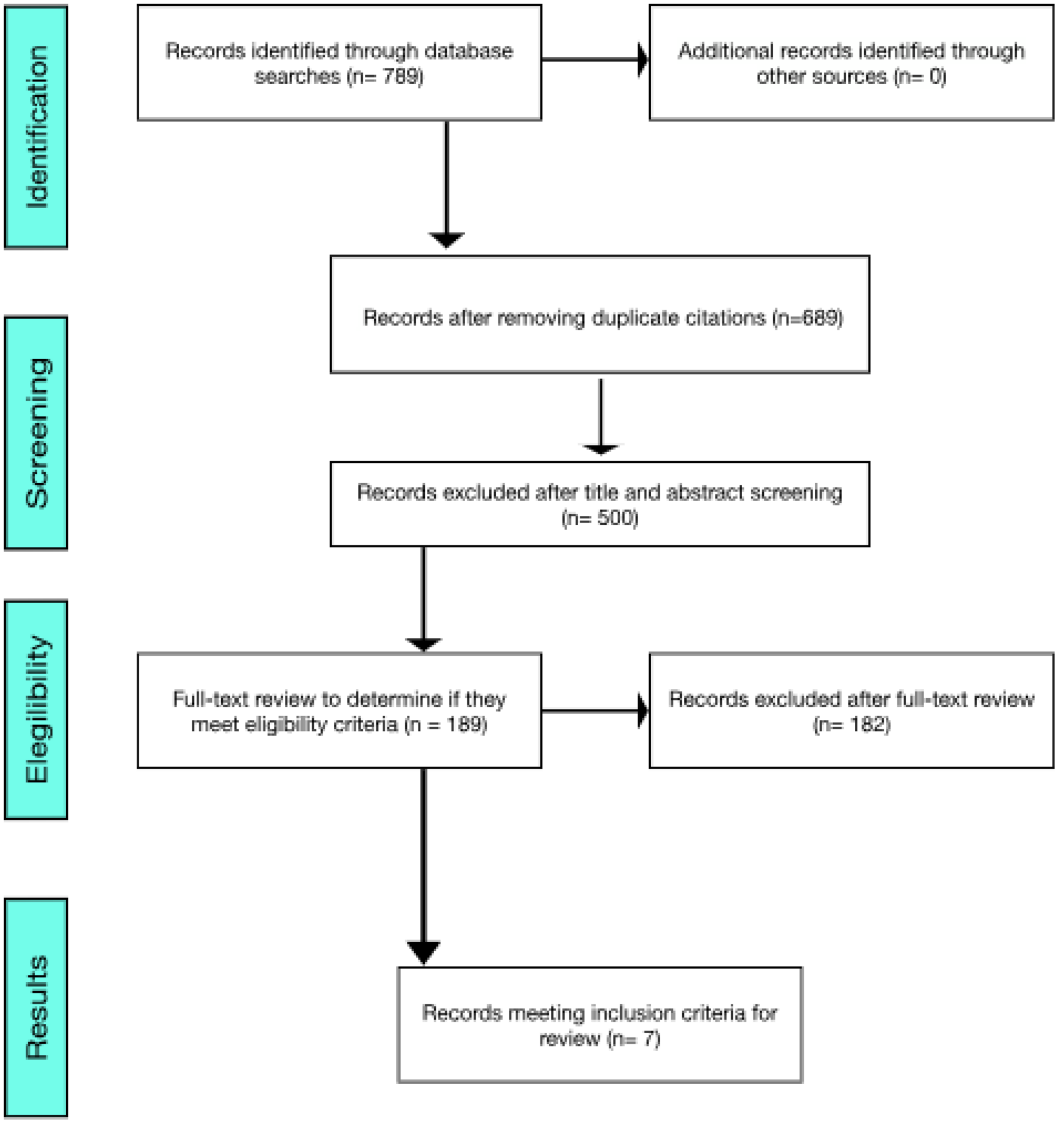
PRISMA diagram of the article selection process. Flow diagram summarizing the different steps carried out based on the model by Moher et al. (2009).

The study selection process was rigorous and systematic, strictly following PRISMA guidelines to ensure the inclusion of relevant, high-quality research. The steps are shown in Figure 1.

### Data analysis

To analyze the studies, an exhaustive review of the seven selected investigations was conducted. Data extraction was carried out using a standardized form, which included the following variables: general study information (author, year of publication, country); study design; population characteristics (type of healthcare professionals, sample size); identified risk factors; and outcomes related to suicidal ideation.

#### Quality assessment

The Newcastle–Ottawa Scale (Wells et al., 2000) was used to assess the methodological quality of observational studies in medical and health research. This tool evaluates how the study was designed and conducted, as well as how potential biases that could influence the results were managed. Each study was rated based on sample selection (1 to 4 points), group comparability (1 to 3 points), and exposure measurement (0 to 2 points). Studies scoring high across all domains were categorized as high quality (7 to 9 points), those with intermediate scores as medium quality (5 to 6 points), and those with lower scores as low quality (0 to 4 points). This classification allowed for the identification of the most notable studies and ensured that the review’s findings were based on high-quality scientific literature.

## Results

The systematic review incorporated a total of seven studies that investigated the prevalence and risk factors associated with suicidal ideation among healthcare personnel. The key characteristics of these studies are summarized in Table 1. Following the examination of the seven studies identified, the relevant data are presented in a summarized and integrated format in Table 3.

### Study characteristics

The studies were conducted in diverse geographical contexts, including Brazil, Türkiye, the United States, Belgium, Spain, Portugal, and India, with publication dates spanning from 2020 to 2024. Most studies utilized a cross-sectional design, allowing for a snapshot of the mental health status of healthcare workers at a specific time, while Mortier et al. (2024) conducted a cohort study to examine trends over time among active healthcare workers during the COVID-19 pandemic.

### Sample size

**Figure 2:**
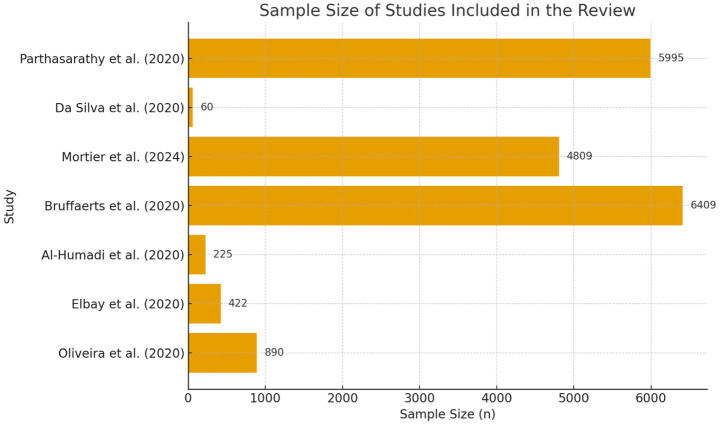
Sample size of studies included in the systematic review. This figure summarizes the sample size (*n*) of each study included in the systematic review, identified by author and year of publication. It visually represents the variability in the number of healthcare professionals analyzed across studies, which contributes to understanding the heterogeneity of the reviewed evidence.

The sample sizes varied significantly across the studies, reflecting the scope and scale of each investigation (see Figure 2). The largest sample was reported by Bruffaerts et al. (2020), which included 6,409 healthcare professionals, highlighting the widespread nature of the issue. Conversely, da Silva et al. (2020) focused on a smaller group of 60 nursing students, offering insight into the unique challenges faced by individuals in training.

### Prevalence of suicidal ideation

While the studies primarily focused on identifying risk factors, they collectively underscored a concerning prevalence of suicidal ideation among healthcare personnel. For instance, Elbay et al. (2020) found that anxiety, stress, and depression were significantly heightened in frontline healthcare workers during the COVID-19 pandemic, correlating with increased suicidal thoughts. This finding is particularly alarming given the added pressures faced by healthcare workers during public health crises.

### Identified risk factors

The review revealed several prominent risk factors associated with suicidal ideation, categorized into occupational, personal, and organizational domains:

- Occupational stressors: A common theme across multiple studies was the impact of prolonged working hours and high patient caseloads. Elbay et al. (2020) reported that healthcare workers with longer shifts and a greater number of COVID-19 patients tended to experience higher levels of anxiety and depressive symptoms, which correlated with suicidal ideation. Mortier et al. (2024) echoed this finding, noting that extended work hours and chronic stress significantly contributed to mental health challenges in healthcare workers.
- Mental health conditions: The presence of underlying mental health issues such as depression and anxiety emerged as critical risk factors. Al-Humadi et al. (2020) found a significant relationship between burnout and suicidal ideation among physicians, emphasizing the importance of recognizing and addressing mental health concerns within this population. Similarly, de Oliveira et al. (2020) highlighted that issues such as anxiety, depression, and insomnia were prevalent among nursing professionals, suggesting a need for mental health support tailored to their specific challenges.
- Financial stress: Financial difficulties were identified as a significant risk factor, particularly among students and early-career professionals. da Silva et al. (2020) reported that nursing students faced challenges related to financial burdens, relocation for studies, and the pressure of high academic expectations, all of which contributed to their risk of suicidal thoughts. This finding underscores the importance of considering socioeconomic factors when addressing mental health in healthcare settings.
- Substance use and PTSD: Substance use was another critical risk factor identified in the literature. Bruffaerts et al. (2020) linked substance use to both suicidal ideation and behaviors, indicating that healthcare workers may resort to unhealthy coping mechanisms in response to occupational stress. The study also highlighted the prevalence of post-traumatic stress disorder (PTSD) among healthcare workers, further complicating their mental health landscape.
- Demographic factors: Specific demographic characteristics, such as medical specialty and duty schedules, were found to influence suicidal ideation. Al-Humadi et al. (2020) noted that certain specialties with higher stress levels were associated with increased rates of suicidal thoughts, suggesting the need for specialized interventions targeting these high-risk groups.

#### Methodological considerations

The studies selected demonstrated high methodological quality according to the Newcastle–Ottawa Scale. Most studies received scores of 7 or higher, ensuring the reliability of the results (see Table 4). In assessing the quality of the studies included, it is essential to note the methodological diversity. The use of standardized assessment tools across studies provided a reliable framework for evaluating mental health outcomes, although variations in sample populations and settings may limit the generalizability of findings. In conclusion, the findings from this systematic review underscore the multifaceted nature of suicidal ideation among healthcare personnel, with significant contributions from occupational stressors, mental health conditions, financial pressures, and substance use. These insights highlight the urgent need for targeted interventions, comprehensive mental health support, and organizational policies that prioritize the well-being of healthcare workers to mitigate these risks effectively (see Table 5).

**Table 4:** Characteristics of the trials used for the review.

Author and year	Country	Study	Design	Population	Sample	Risk factors
de Oliveira et al. (2020)	Brazil	Prevalence and associated factors of suicidal ideation among nursing professionals	Cross-sectional	Nurses	890	Work-related stress, financial problems, depression, anxiety, insomnia
Elbay et al. (2020)	Türkiye	Anxiety, stress, and depression in healthcare personnel during COVID-19	Cross-sectional	Frontline healthcare workers	422	Extended shifts, higher number of COVID patients treated, lower support from peers and supervisors
Al-Humadi et al. (2020)	United States	Depression, suicidal thoughts, and burnout in physicians	Cross-sectional	Physicians	225	Medical specialty, number of recent on-call shifts, history of anxiety and/or depression
Bruffaerts et al. (2020)	Belgium	Suicidal thoughts and behaviors in healthcare personnel	Cross-sectional	Healthcare workers	6,409	Substance use, post-traumatic stress disorder
Mortier et al. (2024)	Spain	Mental health conditions among active healthcare workers during COVID-19	Cohort	Healthcare workers	4,809	Extended shifts, stress, depression, use of anxiolytics
da Silva et al. (2020)	Portugal	Risk factors and suicidal ideation	Cross-sectional	Nursing Students	60	Financial difficulties, relocating to study, high levels of responsibility and pressure
Parthasarathy et al. (2020)	India	Suicidal tendencies, depression, anxiety	Cross-sectional	Healthcare workers	5,995	Anxiety, depression, substance use, lifestyle, family functioning, high responsibility

**Table 5:** Quality assessment: Newcastle–Ottawa Scale.

Author and year	Selection (1–4)	Exposure (0–2)	Comparability (1–3)	Total
de Oliveira et al. (2020)	3	2	2	7
Elbay et al. (2020)	3	1	2	6
Al-Humadi et al. (2020)	4	2	2	8
Bruffaerts et al. (2020)	4	2	2	8
Mortier et al. (2024)	4	2	3	9
da Silva et al. (2020)	2	2	2	6
Parthasarathy et al. (2020)	4	1	2	7

## Discussion

The findings of this review address the main research question, which aimed to identify the risk and protective factors associated with suicidal ideation among healthcare professionals. The systematic review has identified a range of risk factors significantly associated with suicidal ideation among healthcare professionals. Notable among these factors are job stress, lack of social support, depression, anxiety, and burnout (de Oliveira et al., 2020). All of these elements are interrelated and are exacerbated by the personal and professional conditions that healthcare workers face.

Job stress has emerged as one of the most critical factors in suicidal ideation (Elbay et al., 2020). This phenomenon is not surprising, as the healthcare environment is often characterized by high workloads, long hours, rotating shifts, and constant exposure to high-pressure situations, all contributing to significant physical and emotional exhaustion. The studies reviewed make it evident that stress is a precursor to burnout, which is directly linked to suicidal ideation. These findings highlight the need for a deeper focus on managing psychological distress in the workplace to prevent severe consequences such as suicidal thoughts.

Another significant factor identified is the lack of social support in the workplace (Elbay et al., 2020). Healthcare professionals who lack adequate support from colleagues and supervisors may experience a sense of isolation, increasing the risk of suicidal ideation. The absence of a supportive environment heightens stress and also reduces individuals’ ability to handle the emotional demands of their work. This finding aligns with the literature emphasizing the importance of a social network in mitigating stress and promoting mental well-being. Institutions should foster a collaborative and supportive work environment to reduce the risk of isolation and its impact on health.

The review also identified depression and anxiety as prevalent conditions among healthcare professionals that are closely linked to suicidal ideation (Mortier et al., 2024). Some studies indicate that healthcare professionals with mood disorder symptoms are more prone to experience suicidal thoughts. Constant pressure, emotional demands, and the inherent perfectionism of healthcare work contribute to the emergence of these disorders. Evidence suggests that depression and anxiety are not only responses to job stress but also conditioned by a lack of social support and burnout, creating a dangerous cycle that can lead to suicidal ideation. This connection underscores the need for early and effective interventions, including the detection and treatment of these disorders among healthcare professionals.

Burnout, characterized by emotional exhaustion, depersonalization, and reduced personal accomplishment, has been identified as a factor in the occurrence of suicidal thoughts due to unmanaged chronic job stress. The studies in this review have demonstrated a significant correlation between burnout and suicidal ideation (Al-Humadi et al., 2020). Therefore, interventions should focus not only on reducing workload and improving work conditions but also on providing psychological support and wellness programs that address the emotional and physical needs of healthcare professionals.

While common factors were identified in the review, considerable variability in the results among studies was also observed. Factors such as age, gender, and role within the healthcare sector showed differences in their association with suicidal ideation (Parthasarathy et al., 2020). For example, some studies found that women have a higher risk of suicidal ideation than men, while others suggested the opposite. This discrepancy could be due to differences in study methodologies, evaluated populations, or specific cultural and workplace contexts. This variability highlights the need for further research to better understand how these sociodemographic factors influence suicidal thoughts in different subgroups within the healthcare sector.

To further address the variability observed across studies, it is important to note that suicidal ideation manifests differently among subgroups of healthcare professionals. Evidence indicates that physicians tend to present higher rates of suicidal ideation compared to other healthcare workers, often related to professional isolation, high responsibility, and stigma surrounding mental health help-seeking (Hawton et al., 2004). In contrast, nurses are more prone to suicidal ideation associated with emotional exhaustion, heavy workloads, and moral distress due to constant patient care demands and limited institutional support (Davidson et al., 2020). Medical residents and interns also represent a particularly vulnerable group, as long working hours, sleep deprivation, and intense performance pressure contribute to elevated psychological distress (Shanafelt et al., 2022). Meanwhile, allied health professionals—such as therapists and technicians—experience suicidal thoughts primarily linked to job insecurity and lack of recognition. These distinctions emphasize that suicidal ideation is not homogeneous across the healthcare workforce but varies depending on professional role, workplace conditions, and access to support resources. Recognizing these differences is essential for designing targeted interventions and preventive strategies that address the specific needs and stressors of each professional group.

Furthermore, it is important to emphasize the magnitude of the total sample analyzed across the reviewed studies, which collectively included more than 19,000 healthcare professionals from various countries and occupational roles. This large cumulative sample size strengthens the reliability and generalizability of the results, providing a broader understanding of suicidal ideation within this population. However, the variability in sample sizes among individual studies should be taken into account when interpreting the findings, as smaller samples may reduce statistical power and the representativeness of specific subgroups.

Besides risk factors, the review has underscored the importance of protective factors, especially resilience, defined as the ability to adapt positively to adversity, which has been shown to be important in protecting against suicidal ideation (Arioli et al., 2018). Healthcare professionals with high levels of this skill are less likely to experience such thoughts, even when facing high levels of stress and distress. Promoting resilience through training and support programs can be an effective strategy to reduce the risk of suicide in this group. This proactive approach may include developing coping skills, enhancing self-confidence, and promoting a work environment that supports personal and professional growth.

The reviewed literature suggests various intervention strategies that could effectively mitigate the identified risks. These include psychological support programs or policies that reduce workload and increase social backing. Additionally, Joiner’s interpersonal theory of suicide, which focuses on decreasing hopelessness and thwarted belongingness, has been shown to be effective. This therapy addresses factors contributing to suicidal thoughts for crisis intervention (Smith et al., 2022).

Emerging technology also represents an underutilized resource in this field. Mobile apps and digital platforms can provide quick access to mental health resources such as mindfulness, peer support, and symptom monitoring (Lamb et al., 2022), which is particularly useful for healthcare staff facing long shifts. Using these digital tools could represent an effective and accessible way to mitigate suicidal ideation risk and promote resilience in this workforce.

This review presents some limitations that should be considered. One major limitation is the lack of studies with a more detailed gender perspective. While some studies indicate that women experience more suicidal ideation, it is men who tend to carry out more suicidal behaviors (Barroso, 2019). This aspect has not been sufficiently addressed in most of the reviewed studies, limiting the ability to understand how gender influences various aspects of suicidal ideation in healthcare workers.

Another important limitation is the relative scarcity of studies that delve into the role of resilience as a moderator of suicidal ideation risk (Ide et al., 2022). Although its importance has been recognized, the lack of detailed research on this topic limits the understanding of how resilience can be used effectively in specific interventions. The predominant focus on risk factors rather than protective factors leaves a gap in knowledge that should be addressed in future research.

For future studies, it is essential to deepen the study of resilience and how it can counteract the negative effects of job stress, anxiety, and burnout on suicidal ideation. Longitudinal studies are recommended to explore resilience development and strengthening over time and its impact on healthcare professionals’ mental health. Additionally, it is necessary to further investigate the role of gender in suicidal ideation and behaviors, differentiating between risk factors specific to men and women, as well as possible tailored interventions that might be more effective for each group.

Another research avenue deserving attention is the long-term impact of the COVID-19 pandemic on the mental health of healthcare staff. Although initial studies have been conducted, long-term follow-up is essential to assess how the pandemic’s aftermath, such as post-traumatic stress and persistent burnout, will affect suicidal ideation and the overall well-being of these professionals in the coming years.

Finally, exploring how digital technologies, including mental health apps and telemedicine platforms, can be integrated into intervention programs to foster resilience and reduce suicidal ideation is necessary. Comparing traditional interventions with emerging technologies would provide valuable data on the best ways to support healthcare personnel’s mental and emotional well-being, especially in times of crisis and high work demand.

This review consolidates previous evidence on occupational stress, depression, and burnout as major risk factors for suicidal ideation in healthcare professionals, but adds new insights by differentiating these risks across subgroups (physicians, nurses, residents, and allied staff) and by emphasizing the protective role of resilience.

## Conclusion

Preventing suicidal ideation among healthcare professionals is essential not only for safeguarding individual well-being but also for ensuring the long-term sustainability and effectiveness of healthcare systems. Occupational factors—such as excessive workload, limited autonomy, and chronic exposure to high-pressure environments—emerge as primary contributors to burnout and psychological distress. Moreover, personal vulnerabilities, including perfectionism and pre-existing mental health disorders, further heighten the risk of suicidal ideation within this population.

Institutional and organizational efforts should therefore focus on improving working conditions, enhancing psychological support, and fostering a culture of self-care and open communication. Evidence-based interventions, such as cognitive-behavioral therapy and structured wellness programs, can play a pivotal role in mitigating stress and promoting emotional resilience. At the policy level, strategies aimed at regulating work hours, ensuring adequate staffing, and promoting mental health awareness are critical components for systemic change.

This review underscores the urgent need for targeted preventive strategies tailored to the unique challenges faced by healthcare professionals. Priority should be given to workload management, peer support initiatives, and resilience-building programs that strengthen coping mechanisms and reduce vulnerability to suicidal thoughts. Future research should continue to explore gender- and role-specific differences, as well as the long-term effects of organizational support and mental health interventions through longitudinal and interventional studies.

## Data Availability

The datasets presented in this study can be found in online repositories. The names of the repository/repositories and accession number(s) can be found at: retrieved from https://osf.io/zpwjs.
